# Industry-Sponsored Research: Author's Reply

**DOI:** 10.1371/journal.pmed.0030464

**Published:** 2006-10-31

**Authors:** Julio Sotelo

In his letter [1] Dr. Mansfield has raised a debatable issue: “If the CRC [Collegiate Research Council] is a single international monopoly how could the risk of corruption and inefficiency be managed?” By no means would the CRC be an international monopoly; in the proposal it was stated that the CRC would be certified by the appropriate health authorities of any country. Moreover, several CRCs could be established, as long as the participants were nominated by prestigious academic institutions and the CRC was certified by the federal regulatory agencies.

The risk of corruption and inefficiency would be remote for the following reasons. The constituents of the CRC would be distinguished scholars, appointed independently by leading medical institutions and universities. Contrary to Dr. Mansfield's and Dr. Prabhakar's fears [1,2], this council would not require a substantial infrastructure, since its main goal would be to meet periodically, to select the investigators and institutions that would conduct the clinical protocol submitted during that period, and to review the results generated by previous trials. This task could be expedited through the peer review method. The administrative implementation necessary to carry out the decisions of the CRC might be done by existing institutions (e.g., the US Food and Drug Administration), without the formation of an additional bureaucratic body. In this way, the CRC would be strictly maintained as an academic and scientific core whose opinions and sanctions were respected by the individuals and institutions involved in the execution of any given protocol of drug testing.

The CRC would not have “bureaucratic bodies,” as it would function only as a collegiate board to select who would do the testing and where a given protocol of drug testing would be conducted. The research funds provided by the pharmaceutical company would not be administered by the CRC, but by the corresponding institution in charge of the specific research or by the administrators appointed by the CRC.

To the question of who would fund the CRC the answer is simple: a very small percentage of the total cost of the protocol would be used to create a fund to cover travel expenses and stipends for the scholars participating in the CRC.

In response to the statement that the CRC “will consume significant amounts of time and human resources” [2], I do not see why this should be the case. I believe that the members of the CRC could meet once or twice a month and propose, in a single day, one or various candidates for a given protocol. The operation of the CRC would be somewhat similar to that of the editorial board of scientific journals who meet periodically to select reviewers for scientific papers and to evaluate the results of reviews. Once the scholars of the CRC had selected the potential investigators or institutions they would be invited to conduct the research; this task could be accomplished by commissioned personnel independent from the regulatory agency.

With regard to the prices of drugs, I think that Dr. Prabhakar has not chosen a comparable example. In fact, hospital charges and physicians' consultations are not controlled in most countries; that is because these prices are usually set—very much as for many other services and goods—by production costs and competition. As mentioned in my proposal, this is not the case for pharmaceutical substances.
